# Effect on amino acid and mineral content of the loach (*Misgurnus anguillicaudatus*) by adding Fe (II) chelating hairtail protein hydrolysates (Fe (II)‐HPH) to the feed

**DOI:** 10.1002/fsn3.1444

**Published:** 2020-02-05

**Authors:** Jiayu Miao, Huimin Lin, Shan Zhang, Jiancong Huo, Shanggui Deng

**Affiliations:** ^1^ Zhejiang Provincial Key Laboratory of Health Risk Factors for Seafood College of Food and Pharmacy Zhejiang Ocean University Zhoushan China

**Keywords:** amino acid, Fe (II)‐HPH, loach (*Misgurnus anguillicaudatus*), mineral content, nutritional value

## Abstract

To study the effect on amino acid and mineral content of the loach meat by adding Fe (II) chelating hairtail protein hydrolysates (Fe (II)‐HPH) to the feed. A total of 100 healthy loaches were selected. After 1 week's adaptive feeding, they were randomly divided into five groups and fed with feeds containing of Fe (II)‐HPH (0, 0.5, 1, 2, and 4 g/kg). On the 40th day, detection work of general nutrients (moisture, ash, crude protein, and crude fat), mineral elements (Fe, Mn, Cu, Zn, Na, K, and Ca), amino acid and amino acid score (AAS), Chemical Score (CS) and essential amino acid index (EAAI) indexes were done. The results show that crude protein has the highest content while crude fat has the lowest when amount of added Fe (II)‐HPH in feed is 2 g/kg. The Fe content is significantly improved while amount of added is 1, 2, 4 g/kg. The Ca content is significantly improved and the Zn content is significantly improved while amount of added was 2 g/kg. Mn contents are significantly lower than control while amount of added is 4 g/kg. Based on analysis of amino acids in each group, the nutritional value of loach meat with 2 g/kg Fe (II)‐HPH addition amount is relatively high, total amount of essential amino acids increases significantly, and EAA/TAA and EAA/NEAA improve significantly. In conclusion, adding 2 g/kg Fe (II)‐HPH to feed could improve the nutritional values of loach meat.

## Graphical abstract

Fe (II)‐HPH has some influence on amino acid and mineral content of loach meat. Fe, Ca, and Zn content increase when Fe (II)‐HPH amount is 2 g/kg. Fe (II)‐HPH can increase Leu, Ile, and Lys content and improves EAA/TAA and EAA/NEAA of loach meat.

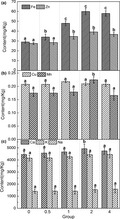

## 1 INTRODUCTION

Loach (*Misgurnus anguillicaudatus*) belongs to order *Crrpiniformes*, family *Cobitidae*, *Misgurnus*. It is a small commercial fish in low habitat (Kitagawa, Fujii, & Koizumi, 2018). Loach contains high‐quality protein, vitamins. It is delicious and nutritious. It also has medical value, for example, loach peptide has antioxidant activities and antifatigue effect and loach polysaccharide could protect the rat hepatocytes against the oxidative damage induced by hydroperoxide in vitro (Wang, Pei, Liu, & Zhou, 2017; You, Zhao, Regenstein, & Ren, 2011).

Iron is an essential trace element for fish growth and is very important in fish bone growth, nerve development, and immunity enhancement (Kundu et al., 2017). Under natural conditions, fish can obtain iron from natural foods and can also absorb water‐soluble iron through the sputum and intestinal mucosa without iron deficiency. But in the commercialized intensive system, feed is almost the only iron source for fish. If iron deficiency is insufficient or iron utilization is adversely affected, this may lead to iron deficiency in fish, slowing its growth and make its ability of preventing disease and nutritional value decline (Guo et al., 2018). In recent years, some scholars have discovered that peptides produced by hydrolysis of natural proteins can be combined with trace metal elements (Chen et al., 2019; Wu et al., 2019). Some of those peptides greatly promote the body's absorption of iron, while avoiding the disadvantages of low absorption and utilization of traditional iron supplements, and certain toxic side effect (O'Loughlin, Kelly, Murray, FitzGerald, & Brodkorb, 2015; Takeda et al., 2016), and also has activity such as antioxidation and antibacterial, and thus has received extensive attention (Fang, Xu, Lin, Cai, & Wang, 2019; Lin, Tang, Xu, & Wang, 2019). Lin, Deng, and Huang (2014) studied the preparation process of ferrous chelating peptide using complex enzymatic hydrolysis hairtail (*Trichiurus lepturus*) protein and adopted ultrafiltration method to prepare high‐antibacterial and antioxidant Fe(II)‐HPH. Huang, Lin, and Deng (2015) studied the infrared spectra of chelation product from the peptides of different molecular weights of fish enzymatic hydrolysate and Fe^2+^ and their antianemia and antifatigue mechanisms in anemia model rats. They found that Fe (II)‐HPH has the effect of improving the symptoms of anemia in rats and at the same time regulating the intestinal microflora of rats (Lin, Deng, Huang, Li, & Song, 2016). Zhang, Shi, Wang, and Deng (2016) found that adding the right amount of peptide‐Fe^2+^ complex to the feed would increase the growth performance of *Procambarus clarkii* and other nonspecific immune enzymatic activities.

At present, the related research on Fe (II)‐HPH on loach has not been reported. This research aims to study the influence on the nutritional value of loach meat by adding prepared Fe (II)‐HPH in feed. Firstly, 50 healthy loaches were selected, and after 1 week's adaptive feeding, they were randomly divided into five groups and fed with feeds containing of Fe (II)‐HPH (0, 0.5, 1, 2, and 4 g/kg). Secondly, feeding lasted for 40 days and measured the general nutrients (moisture, ash, crude protein, crude fat), mineral elements (Fe, Mn, Cu, Zn, Na, K, Ca), amino acid composition. Finally, evaluate the amino acid score (AAS), Chemical Score (CS), and essential amino acid index (EAAI) of loach muscle.

## 2 MATERIALS AND METHODS

### 2.1 Materials and equipment

Live Loaches (*Misgurnus anguillicaudatus*) were bought in Zhoushan Nanzhen Market. Each has a body weight of 15 ± 1.5 g and a body length of 13 ± 1.0 cm and was sent to the laboratory by plastic water tank. Hairtail surimi was provided by Zhejiang Xingye Ltd. Ferrous chloride and papain were made from Sinopharm Chemical Reagent Co., Ltd.

Preparation of hairtail enzymatic peptide (Lin et al., 2014): The hairtail surimi (stored at −20°C) were thawed in a 4°C refrigerator overnight, then were evenly distributed after homogenization with deionized water at a ratio of 1:10 (g:ml). pH was adjusted to 6.5, and then, the Papain was added to homogenate in proportion of 20,000 U/g for 8 hr at 45°C. pH was adjusted with 1 mol/L NaCl and 1 mol/L HCl in the reaction to maintain it at 6.5 throughout the hydrolysis process. After reaction was completed, it was placed in a 95°C water bath, inactivated enzyme for 20 min, cooled to room temperature, obtained the supernatant liquid after precipitation, and then used an ultrafiltration membrane of 5 k Da to remove macromolecular peptides.

#### 2.1.1 Chelation reaction

Enzymatic peptide solution which molecular weight is <5 kDa was added with erythorbic acid at concentration of 1%, well mixed, then was added with 2% ferrous chloride solution at concentration of 1 mol/ml of total volume, pH was adjusted to 4.0 and oscillated for 30 min with 30°C water bath and then centrifuged at 1,000 *g* at 4°C for 20 min. The supernatant which is Fe (II)‐HPH was obtained, and then, it was evaporated (40°C, 4 hr) and freeze‐dried (−50°C, 36 hr) to a powder for later use.

#### 2.1.2 Feed preparation

The experimental feed was prepared as described by Chang et al. (2012), and the formulations were shown in Table 1. The experimental feed was pulverized by a Chinese herbal medicine pulverizer (JX‐NNJ‐1000G, Guangzhou Degong Machinery Equipment Co., Ltd.), and Fe (II)‐HPH was added to the experimental feed at levels of 0 (control group), 0.5, 1, 2, and 4 g/kg. A small feed granulator (120B, Anyang Gemco Machinery Co., Ltd) was used to make the mixture into small particles of the sinking feed and then dried in an oven at 25°C.

**Table 1:** Ingredient and chemical analysis of the basal feed (dry weight) for loach

Ingredient	g/kg
Fish meal	210
Soybean meal	360
Wheat flour	100
Wheat bran	70
Rapeseed meal	40
Peanut oil	20
Corn flour	170
Ca (H_2_PO_4_)_2_	15
Sodium methylcellulose	10
NaCl	3
Vitamin mixture	1.2
Mineral	0.8
Proximate composition (% dry matter)	
Crude protein	30.22
Crude lipid	7.06
Ash	6.67

Note

Vitamin premix provided per kg of diet: VA: 12,500,000 IU; VB1: 800 mg; VB2: 2,500 mg; VB6: 800 mg; VB12: 10 mg; VD: 2,000,000 IU; VE: 7,000 IU; VK: 2,000 mg; niacin: 3,000 mg; pantothenic acid: 10,000 mg; folic acid: 300 mg; biotin: 20 g; VC: 20,000 mg. Mineral provided per kg of diet: Mn:19 mg; Mg: 230 mg; Co: 0.1 mg; I: 0.25 mg; Fe: 100 mg; Cu: 2.5 mg; Zn: 65 mg; Se: 0.2 mg.

### 2.2 Loach breeding

The plastic water tanks (60 cm × 44 cm × 36 cm) were sterilized with a 0.01% potassium permanganate solution. A total of 100 loaches (the whole experiments were repeated twice, 50 loaches for each replicate) were placed in a 1.5% sodium chloride solution, sterilized for 10 min, placed in a plastic tank, and domesticated for 10 days at 25 ± 1°C. After the end of domestication, healthy loaches with no surface damaged were selected and divided into five groups with 10 in each experimental group, then put them into another plastic tank. Feed was placed at 8:30 a.m. and 4:30 p.m. every day, and the amount of feed is 1%–2% of the weight of loach. Remaining amount should be sucked out in time. Water temperature was 25 ± 1°C, and daily water exchange was about 1/3. Feeding lasted for 40 days and was stopped 1 day before the end of the feeding. After the end of feeding, every loach was dissected and the meat was removed, minced and placed in a −80°C refrigerator for storage.

### 2.3 Determination of general nutrients

Ash measurement was measured using a 550°C dry ash method (Liu, 2019). Loach meat moisture content was determined by oven drying the muscle samples at 105°C until constant weight (about 12 hr).Total fat was extracted from 10 g thawed loach meat with methanol and chloroform (1:1, v:v) according to the Sahena et al. (2010). Total protein content of loach meat was measured by the Kjeldahl method (Chromy, Vinklárková, Šprongl, & Bittová, 2015). The titration was done with a standardized hydrochloric acid (0.1 mol/L) until a pH of 6.10 was reached. To calculate protein from nitrogen, the factor 6.25 was used (Sriperm, Pesti, & Tillman, 2011). Each assay was carried out in triplicate.

### 2.4 Determination of mineral elements in meat

1.0 g thawed meat was added to the digestive tract, and then, concentrated nitric acid and perchloric acid were added in a volume ratio of 4:1 to 15 ml, mixed uniformly, and placed on a digestion furnace to be heated and digested. It was heated at 130°C for 1 hr, warmed to 150°C for 5 hr, and then warmed to 180°C for 1 hr, and finally, the temperature was raised to 210°C, the lid of the digestive tract was opened, concentrated nitric acid was added, and the concentration was concentrated to about 2 ml. After it was cooled to room temperature, it was made up to a suitable volume and measured by an atomic absorption spectrometer (AA‐7000 atom absorption, Shimadzu Japan).

### 2.5 Amino acid analysis

Amino acid compositions of loach meat were analyzed using an amino acid analyzer (L‐8900, Hitachi) according to Kasozi, Iwe, Sadik, Asizua, and Namulawa (2019) with some modifications. Briefly, thawed loach meat was hydrolyzed in 6 M HCl solution with drops of phenol for 24 hr at 110°C after 10 min of nitrogen blowing. Then, 1 ml hydrolysis was centrifuged at 6,000 *g* for 5 min and 200 μl of supernatant was evaporated by nitrogen blowing at 50°C. The residual was dissolved in 1.5 ml of 0.2 M HCl solution and passed through a 0.45 μm membrane filter. Twenty microliters of the hydrolysis were injected using an auto‐sampler. Mixed standard amino acids with taurine standard were analyzed before sampling. The amino acids were identified and quantified by comparing peak profiles of the squid samples with standard amino acid profiles. On the basis of the amino acid composition, amino acid score (AAS), Chemical Score (CS), and essential amino acid index (EAAI) were calculated according to Rosa and Nunes (2004).

### 2.6 Statistical analysis

The whole experiments were repeated twice and each loach was assigned for a specific treatment. Analyses were conducted in triplicate for each replicate experiment. Statistical analysis was performed by SPSS software, version 17.0 (SPSS Statistical Software, Inc.). The significance was defined at *p* < .05.

## 3 RESULTS AND DISCUSSION

### 3.1 General nutrients in loach meat

Table 2 shows the measurement result of general nutrients in loach meat. There is no significant difference (*p* > .05) in moisture, ash, and crude fat between experimental group and control group. Crude protein is a little high in the group where the add‐in amount of Fe (II)‐HPH is 2 g/kg. Zhao, Zhao, and Lv (2000) found that after adding amino acid chelating salts to the feed, there was no significant different in the basic nutrients of tilapia and carp, but the ratio of protein was a little high which is accordance with our results. Due to higher bioavailability compared to inorganic salts, chelated minerals as feed supplements have beneficial effects on growth performance or immunity for animal (Apines‐Amar et al., 2004; Manangi, Vazques‐Añon, Richards, Carter, & Knight, 2015; Sarker, Satoh, & Kiron, 2007). Dietary iron levels significantly influenced whole body moisture, protein, and lipid content (Musharraf & Khan, 2019). We speculated that the crude protein in loach meat increased because Fe (II)‐HPH was used as additive to supplement amino acids in feed (Eckert et al., 2016) and the increase in whole body protein also may be because of the availability of optimal amount of iron activating the protein metabolism resulting into improved protein retention and fish growth (Musharraf & Khan, 2019). But when the concentration of Fe (II)‐HPH was 4 g/kg, the proportion of crude protein and crude fat in loach meat decreased, which may be due to the excess iron levels in the diet depressed fish growth (Lall & Dumas, 2015).

**Table 2:** General nutritional composition of loach (*Misgurnus anguillicausatus*) meat (dry weight)

Group	Moisture	Ash	Crude fat	Crude protein
0	73.31 ± 0.20a	0.92 ± 0.14a	7.03 ± 0.07a	18.76 ± 0.44b
0.5	73.92 ± 0.42a	0.96 ± 0.17a	6.69 ± 0.07a	18.71 ± 0.41b
1	72.99 ± 0.23a	0.91 ± 0.11a	6.81 ± 0.15a	18.81 ± 0.05b
2	72.93 ± 0.49a	0.90 ± 0.05a	6.42 ± 0.48a	19.34 ± 0.14c
4	73.39 ± 0.41a	0.83 ± 0.05a	6.93 ± 0.28a	17.74 ± 0.45a

Note

0, 0.5, 1, 2, and 4, respectively, meant the additive Fe(II)‐HPH (g/kg) amount of 0, 0.5, 1, 2, and 4 g/kg in feed; different lower case letters denote significant differences (*p* < .05) between different treatment groups.

### 3.2 Mineral elements in loach meat

**Figure 1:**
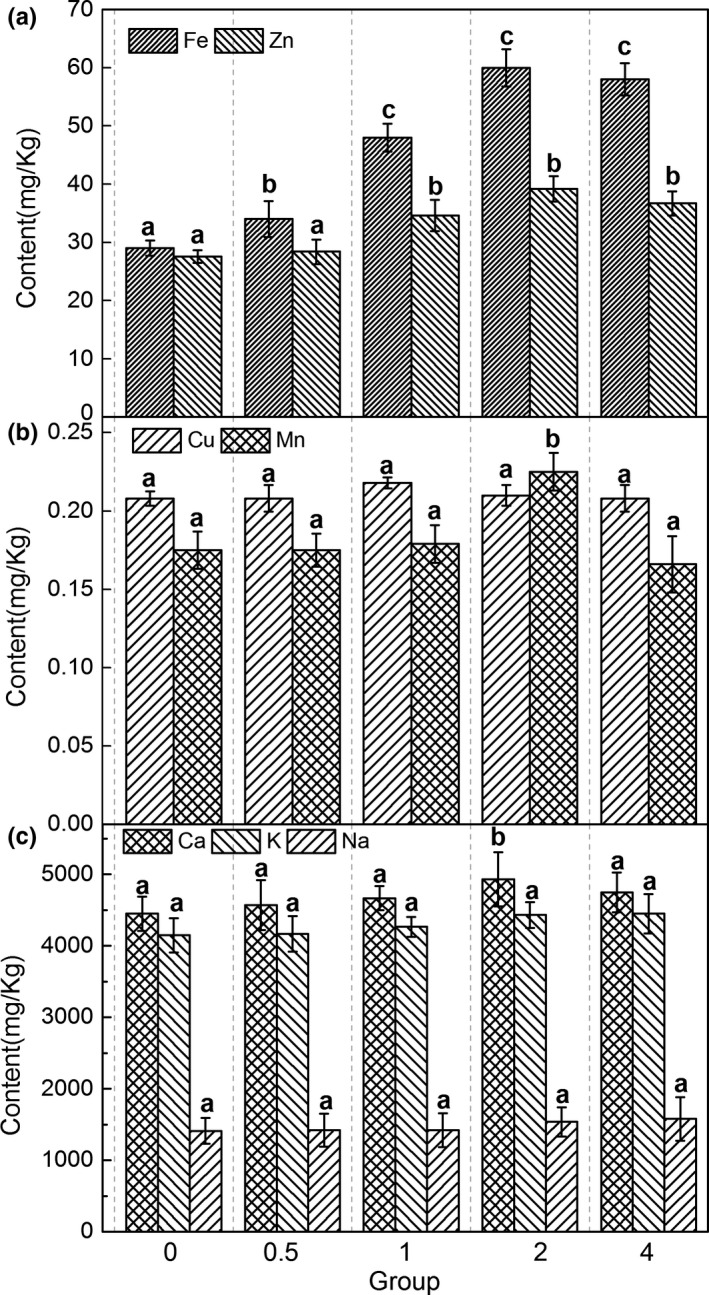
Effect on mineral elements of the loach (*Misgurnus anguillicaudatus*) meat (dry weight) by adding Fe (II)‐HPH to the feed. 0 meant the control group, 0.5, 1, 2, and 4 respectively meant the additive Fe (II)‐HPH amount of 0.5, 1, 2, and 4 g/kg in feed. Different lower case letters denote significant differences (*p* < .05) between different treatment groups

Figure 1 gives the content of mineral elements measured in each experimental group. It can be found that the content of Mn element is extremely significant (*p* < .01) lower than control group when addition amount is 4 g/kg (Figure 1A). Fe is significantly higher (*p* < .05) with addition amount 0.5 g/kg and is extremely significantly higher than control group (*p* < .01) with addition amount 1, 2, and 4 g/kg, and Zn is extremely significant higher (*p* < .01) with addition amount 2 g/kg (Figure 1B). Ca is significantly higher (*p* < .05) with addition amount is 2 g/kg (Figure 1C).There is no significant difference (*p* > .05) in the content of Cu, K, and Na in each experimental group.

In the fish body, mineral element content is small but plays an extremely significant physiological role (Guo et al., 2018; Silva et al., 2020). Na and K can enhance muscle excitation maintain cardiac rhythm and in a certain way participate in protein carbohydrate and heat metabolism improve body vitality and health level (Fujiwara‐Nagata & Eguchi, 2004; Parks, Tresguerres, & Goss, 2008). Mn, Zn, Fe and Cu are auxiliary components of an enzyme or activators of enzyme in the animal body (Wood, 2011). They participate in the construction of organic compounds of certain special functions in tissues and have a direct or indirect effect on the fish body's immune process (Lall, 2003). In this experiment, Fe (II)‐HPH has no significant effect on the content of Cu (Figure 1B), K, and Na (Figure 1C) in loach. Our result is similar to Fischer (Fischer, Giroux, & l'Abbé, 1984), who had reported that addition of iron ameliorated the effects on iron but not on copper status. But when the addition amount of Fe (II)‐HPH is 2 g/kg in loach feed, the content of Ca (Figure 1C), Fe, and Zn (Figure 1A) all are significantly higher (*p* < .05) than those in control group, the content of Mn (Figure 1B) is extremely significantly lower (*p* < .01) (Fe(II)‐HPH 4 g/kg) than that in control group. It has been reported that chelated or organic sources of iron were found to be more available to fish than ferrous sulfate (Antony Jesu Prabhu, Schrama, & Kaushik, 2014). Davidson, Almgren, Sandström, and Hurrell (1995) found that added iron to solid foods had no effect on zinc absorption. But in our result, Fe (II)‐HPH can increase the amount of zinc in loach meat. Varying the levels of iron in oral dosages has been shown to influence the percent of Mn absorbed (Davis, Wolf, & Greger, 1992). In our research, when the concentration of Fe(II)‐HPH reaches 4 g/kg, the content of Mn in loach meat drops significantly. The organic sources of Mn, Zn, and Cu have advantage over the sulfate forms in terms of the blood immunoglobulins (Roshanzamir, Rezaei, & Fazaeli, 2019), so our results indicate that 2 g/kg Fe (II)‐HPH in feed not only promotes the vital function and health level of the loach body but also improves the edible value of loach.

### 3.3 Analysis of the composition and content of amino acid in loach meat

The type and content of amino acids are important factors influencing the nutritional value of proteins. Table 3 shows the type and content of amino acids of loach meat in different groups. Tryptophan was destroyed during the digestion process, and no additional measurements were made. Each group detected 17 amino acids and eight essential amino acids, while the highest amino acid was Glu and the lowest amino acid was Cys in all groups. In essential amino acids, both Leu and Ile are significantly higher than those in control group (*p* < .05) when Fe (II)‐HPH addition amount is 2 g/kg, but only Ile is significantly higher when addition amount is 4 g/kg, and the content of Valand Lys are significantly lower than control group. In nonessential amino acid, Cys and Glu are significantly lower than control group (*p* < .05) when Fe (II)‐HPH addition amount is 2 g/kg. Ser and Ala are significant higher (*p* < .05), and Ala is extremely significant (*p* < .01) when addition amount is 4 g/kg. In each treatment group, total amount of amino acids (TAA), essential amino acids (EAA), and delicious amino acids (DAA) have no significant difference. EAA/TAA ratio is 38%–41%, while EAA/NEAA ratio is 78%–81%. They are both significantly higher than control group when addition amount is 2 g/kg. According to the ideal model of FAO/WHO (FAO, 1990), EAA/TAA and EAA/NEAA are higher than 40% and 60%, respectively, in better protein. Such results showed that adding 2 g/kg Fe (II) ‐ HPH to feed could improve the EAA and protein quality in the loach meat. Asp, Gly, Glu, and Ala are the main factors affecting the delicious taste of meat (Dashdorj, Amna, & Hwang, 2015; Sarower, Hasanuzzaman, Biswas, & Abe, 2012). As the Fe (II)‐HPH is added constantly, the content of DAA decreases, but there is no difference among five groups, indicating that Fe (II)‐HPH does not affect the delicious taste of loach meat. This result indicated that feeding the loach with adding 2 g/kg Fe (II) ‐ HPH to could not affect the taste of loach meat.

**Table 3:** Amino acid content of loach (*Misgurnus anguillicausatus*) meat (dry weight)

Amino acid	Content of Fe(II)‐HPH (g/kg)				
	0	0.5	1	2	4
Ser^a^	2.93 ± 0.05a	2.94 ± 0.06a	2.93 ± 0.03a	2.89 ± 0.02a	3.06 ± 0.08b
Tyr^a^	2.02 ± 0.11a	1.99 ± 0.01a	1.89 ± 0.05a	1.91 ± 0.05a	2.08 ± 0.01a
Pro^a^	2.75 ± 0.05a	2.77 ± 0.01a	2.86 ± 0.08a	2.76 ± 0.08a	2.85 ± 0.03a
Cys^a^	0.42 ± 0.05b	0.42 ± 0.03b	0.41 ± 0.04b	0.31 ± 0.03a	0.43 ± 0.03b
Ala^a^, ^b^	4.19 ± 0.07b	4.20 ± 0.02b	4.13 ± 0.02b	4.18 ± 0.06b	3.92 ± 0.02a
Glu^a^, ^b^	10.92 ± 0.20a	10.95 ± 0.18a	10.88 ± 0.02a	10.52 ± 0.02a	10.7 ± 0.02a
Asp^a^, ^b^	6.94 ± 0.15a	6.81 ± 0.03a	6.89 ± 0.02a	6.86 ± 0.01a	6.97 ± 0.02a
Gly^a^, ^b^	3.28 ± 0.01a	3.26 ± 0.09a	3.29 ± 0.01a	3.38 ± 0.06a	3.30 ± 0.04a
Arg^c^	4.09 ± 0.09a	4.14 ± 0.07a	4.11 ± 0.03a	4.21 ± 0.01a	4.02 ± 0.05a
His^c^	2.08 ± 0.05a	2.10 ± 0.12a	2.19 ± 0.09a	2.00 ± 0.09a	2.15 ± 0.03a
Thr^d^	3.25 ± 0.01a	3.29 ± 0.07a	3.32 ± 0.04a	3.29 ± 0.03a	3.28 ± 0.07a
Phe^d^	2.73 ± 0.08a	2.71 ± 0.01a	2.64 ± 0.04a	2.80 ± 0.06a	2.78 ± 0.07a
Leu^d^	5.62 ± 0.03a	5.61 ± 0.01a	5.67 ± 0.03a	5.82 ± 0.02b	5.59 ± 0.01a
Ile^d^	3.03 ± 0.11a	3.02 ± 0.05a	2.96 ± 0.02a	3.34 ± 0.01c	3.20 ± 0.03b
Val^d^	3.44 ± 0.08b	3.33 ± 0.06b	3.50 ± 0.02b	3.22 ± 0.02a	3.21 ± 0.13a
Met^d^	1.89 ± 0.00a	1.92 ± 0.03a	1.91 ± 0.02a	1.85 ± 0.07a	1.91 ± 0.03a
Lys^d^	6.35 ± 0.10a	6.32 ± 0.03a	6.36 ± 0.04a	6.49 ± 0.01b	6.20 ± 0.01a
TAA	65.98 ± 0.99	65.80 ± 0.51a	65.93 ± 0.21	65.83 ± 0.01	65.58 ± 0.22a
EAA	26.32 ± 0.66a	26.21 ± 0.19a	26.34 ± 0.09a	26.81 ± 0.01b	26.40 ± 0.14a
NEAA	33.46 ± 0.53a	33.35 ± 0.36a	33.29 ± 0.01a	32.81 ± 0.47a	33.22 ± 0.06a
DAA	25.34 ± 0.42a	25.23 ± 0.28a	25.20 ± 0.03a	24.94 ± 0.11a	24.80 ± 0.02
EAA/TAA	38.89 ± 0.07a	39.83 ± 0.01a	39.95 ± 0.02a	40.72 ± 0.16b	40.31 ± 0.35b
EAA/NEAA	78.65 ± 0.21a	78.59 ± 0.27a	79.11 ± 0.30a	81.71 ± 0.86b	79.57 ± 0.57a

Note

Different lower case letters denote significant differences (*p* < .05) between different treatment groups.

Abbreviations: Ala, alanine; Arg, arginine; Asp, aspartic acid; Cys, Cysteine; DAA, total delicious amino acids; EAA, total essential amino acids; Glu, glutamine; Gly, glycine; His, histidine; Ile, isoleucine; Leu, leucine; Lys, lysine; Met, methionine; NEAA, total nonessential amino acid; Phe, phenylalanine; Pro, Proline; Ser, serine; TAA, total amino acids; Thr, threonine; Tyr, tyrosine; Val, valine.

a The nonessential amino acids.

b The delicious amino acid.

c The semiessential amino acid.

d The essential amino acids.

### 3.4 Protein nutrition evaluation in loach meat

The number and composition ratio of essential amino acids are the main factors affecting the nutritional value of food proteins, in which AAS, CS, and EAAI are common indicators of evaluating food nutritional value. Data in tables are multiplied by 0.625 and compared with amino acid scoring mode recommended by FAO/WHO and the amino acid pattern of whole egg protein (FAO, 1990), AAS/CS/EAAI are obtained as Table 4.

**Table 4:** AAS, CS, and EAAI in loach (*Misgurnus anguillicausatus*) meat (dry weight)

	EAA							EAAI
	Ile	Leu	Lys	Met+Cys	Phe+Tyr	Thr	Val	
FAO/WHO standard value	2.5	4.4	3.4	2.2	3.8	2.5	3.1	
AAS								
0	0.76	0.80	1.17	0.66	0.78	0.81	0.69	
0.5	0.76	0.80	1.16	0.66	1.34	0.82	0.67	
1	0.74	0.81	1.17	0.66	1.29	0.83	0.71	
2	0.84	0.83	1.19	0.61	1.34	0.82	0.65	
4	0.80	0.79	1.14	0.66	1.38	0.82	0.65	
Egg protein standard value	3.31	5.34	4.41	3.86	5.65	2.92	4.10	
CS								
0	0.57	0.66	0.90	0.37	0.53	0.70	0.52	58.79
0.5	0.57	0.66	0.90	0.38	0.52	0.70	0.51	58.56
1	0.56	0.66	0.90	0.38	0.50	0.71	0.53	58.65
2	0.63	0.68	0.92	0.35	0.52	0.70	0.49	58.90
4	0.60	0.65	0.89	0.38	0.54	0.70	0.49	58.80

Note

0 meant the control group, 0.5, 1, 2, and 4, respectively, meant the additive Fe (II)‐HPH amount of 0.5, 1, 2, and 4 g/kg in feed.

Met+Cys is the lowest in each group as shown in Table 4, which means the first limiting amino acid of loach is Met+Cys (Jirsa, Salze, Barrows, Davis, & Drawbridge, 2013). Lys has the highest AAS (>1) and has a good complementarity with the protein commonly eaten in cereals. EAAI can access how close the essential amino acid content is to the standard protein (Azi et al., 2019). EAAI values in each group are 58.79, 58.56, 58.65, 58.90, and 58.80. It indicates that Fe (II)‐HPH in the feed will promote the nutritional value of the loach when the addition amount of is 2 g/kg. Lin et al. (2016) studied that principal sites of Fe (II) bonding in Fe(II) chelating peptides are primarily carboxylate groups followed by peptide bonds, so the structure of Fe(II) chelating peptides is more stable. Small peptide chelating trace element, because of its special structure stability, can reduce the formation of free radicals during absorption, enhance sterilizing ability, regulate body immunity, and improve disease resistance; thereby, it can increase animal performance (Chen et al., 2019; Sun et al., 2016; Wu, Li, Hou, Zhang, & Zhao, 2017).

## 4 CONCLUSION

Fe (II)‐HPH has some influence on amino acid and mineral content of loach meat. Crude protein has the highest content while crude fat has the lowest when amount Fe (II)‐HPH adding is 2 g/kg. Fe, Ca, and Zn content increase when Fe (II)‐HPH amount adding is 2 g/kg. Fe (II)‐HPH can increase Leu, Ile, and Lys content and improves EAA/TAA and EAA/NEAA of loach meat. In conclusion, adding 2 g/kg Fe (II)‐HPH to feed could improve the nutritional values of loach meat.

## CONFLICT OF INTEREST

The authors declare that they do not have any conflict of interest.

## ETHICAL APPROVAL

This study was approved by the Ethics Committee of Zhejiang Ocean University, China (IEC 20161224). All procedures were carried out in compliance with relevant laws and institutional guidelines.

## INFORMED CONSENT

Written informed consent was obtained from all study participants.
